# A Compressive Sensing Model for Speeding Up Text Classification

**DOI:** 10.1155/2020/8879795

**Published:** 2020-08-07

**Authors:** Kelin Shen, Peinan Hao, Ran Li

**Affiliations:** ^1^School of Foreign Languages, Xinyang Agriculture and Forestry University, Xinyang 46400, China; ^2^School of Computer and Information Technology, Xinyang Normal University, Xinyang 46400, China; ^3^Henan Key Lab of Analysis and Applications of Education Big Data, Xinyang 46400, China

## Abstract

Text classification plays an important role in various applications of big data by automatically classifying massive text documents. However, high dimensionality and sparsity of text features have presented a challenge to efficient classification. In this paper, we propose a compressive sensing- (CS-) based model to speed up text classification. Using CS to reduce the size of feature space, our model has a low time and space complexity while training a text classifier, and the restricted isometry property (RIP) of CS ensures that pairwise distances between text features can be well preserved in the process of dimensionality reduction. In particular, by structural random matrices (SRMs), CS is free from computation and memory limitations in the construction of random projections. Experimental results demonstrate that CS effectively accelerates the text classification while hardly causing any accuracy loss.

## 1. Introduction

With the advancement of information technology over the last decade, digital resources have penetrated into all fields in our society, generating big data, which present a new challenge to data mining and information retrieval [1]. Texts are very common in daily life, and, with their large numbers, it remains an open question to organize and manage them [2]. As one of the fundamental techniques in natural language processing (NLP), text classification means assigning labels or categories to texts according to the content, and it is key to solving the problem of text overloads [3]. In its broad applications such as sentiment analysis, topic labeling, spam detection, and intent detection, text classification provides support for the efficient query and search of texts, attracting a lot of attention from both academia and industry [4, 5].

Word matching (WM), the simplest method in text classification, determines the category of a text by the categories of most words in the text [6]. But, due to the ambiguity of word meaning, WM fails to provide satisfying accuracy. By representing words as vectors, the vector space model (VSM) [7] improves the accuracy of text classification, thus replacing WM as the popular method, but the model requires many rules and great efforts from professionals in labeling texts, which would be a lot of cost. As machine learning (ML) [8] continues to develop, the accuracy of text classification has been further improved. By extracting features from a text to train a classifier, ML reforms VSM and avoids the rule-based inference. Recently, the rapidly developing deep learning (DL) [9], which is a branch of ML, has made text classification more efficient. However, high dimensionality and sparsity of text features pose a challenge to ML, restricting the practical use of ML-based text classification.

In ML, many classifiers can be used to classify texts, such as support vector machine (SVM) [10], decision tree [11], adaptive boosting (AdaBoost) [12], K-nearest neighbor (KNN) [13], and Naïve Bayes [14]. To train these classifiers, texts must be represented as feature vectors by some feature extraction models, among which the commonest is Bag of Words (BOW) [15]. BOW uses the term frequencies of n-grams in the vocabulary constructed by N-Gram [16] to encode every text. Because vocabulary may potentially run into millions, BOW faces the curse of dimensionality; that is, it produces a sparse representation with a huge dimensionality, resulting in the impracticality of training classifiers. Therefore, dimensionality reduction (DR) is used to reduce the size of feature space. In DR, the most common techniques still introduce some time and memory complexity due to their nature of supervised learning, including principal component analysis (PCA) [17], independent component analysis (ICA) [18], and nonnegative matrix factorization (NMF) [19]. Many DL networks use autoencoder to compress the size of parameters. An autoencoder is a neural network that is trained to attempt to copy its input. Some popular architectures include sparse autoencoder [20], denoising autoencoder [21], and variational autoencoder [22]. Internally, they have a hidden layer that describes a code used to represent the input. By being embedded into the neural network, the autoencoder can end up learning a low-dimensional representation very similar to PCAs.

Compared with the above-mentioned DR techniques, random projection [23, 24] is a better choice, since it avoids the model training, but it is still a challenge to store random projections due to the huge dimensionality of text feature. Compressive sensing (CS) [25–27], which has recently been rapidly developing, can be regarded as a random projection technique specially for sparse vectors, and it proves that the perfect recovery of sparse vector can be realized by several random projections. CS retains the advantages of random projection in DR and further overcomes the problem of memory with the help of structural random matrices (SRMs) [28, 29], which makes CS a potential DR technique for text classification. In view of the merits of CS, we use it to speed up the training of text classifiers in this paper. For a low time and memory complexity, SRMs are selected as CS measurement matrices to reduce the size of sparse feature vector. Experimental results demonstrate that CS effectively accelerates the text classification while hardly causing any accuracy loss.

The rest of this paper is organized as follows.  Section 2 briefly reviews text classification and CS theory.  Section 3 describes the CS model for text classification in detail.  Section 4 presents experimental results, and finally Section 5 concludes this paper.

## 2. Background

### 2.1. Text Classification

Given a text dataset *D* = {*d*_1_, *d*_2_,…, *d*_*L*_} of *L* documents and a set *C* = {*c*_1_, *c*_2_,…, *c*_*J*_} of *J* predefined categories, the goal of text classification is to learn a mapping *f* from inputs *d*_*i*_ ∈ *D* to outputs *c*_*j*_ ∈ *C*. If *J* = 2, it is called binary classification; if *J* > 2, it is called multiclass classification. The mapping *f* is called the classifier, and it is trained by being fed with a labeled dataset, where each document in *D* has been assigned a category from *C* by professionals in advance. The trained classifier *f* is used to make predictions on new documents which are not included in *D*. Because of the subjectivity of text labeling, a test dataset is still needed to evaluate the prediction accuracy of *f*.

**Figure 1:**
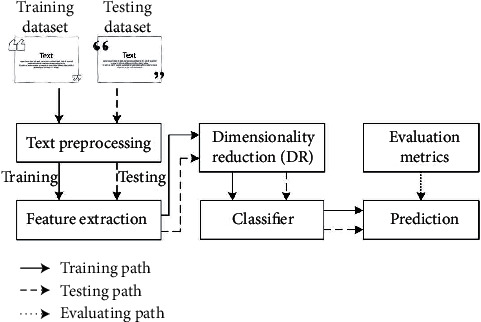
A typical flow of text classification.

A typical flow of text classification is illustrated in Figure 1. In text preprocessing, we tokenize each document in *D*, erase punctuations, and remove unnecessary words such as stop words, misspelling, and slang. To reduce the size of vocabulary from *D*, some operations, e.g., capitalization, lemmatization, and stemming, can also be added. After text preprocessing, feature extraction is performed to represent documents in *D* as feature vectors, which is a crucial step for the accuracy and complexity of text classification. By N-Gram, we collect n-grams from *D* as the vocabulary of BOW model. It is very common to use unigram and bigram, where unigram is a single word and bigram is a word pair. Each document in *D* is encoded as a feature vector based on the frequency distribution of its n-grams on the BOW vocabulary. The size of feature vector is the same as that of BOW vocabulary, resulting in the huge dimensionality of feature space. By using DR techniques, dimensionality can be significantly decreased, reducing the time complexity and memory consumption when training the classifier. The feature vector of a document is also highly sparse because the number of its n-grams is far smaller than the size of BOW vocabulary. The high sparsity makes it possible to realize DR by CS without the loss of classification accuracy. Compared with the traditional DR methods, CS not only avoids the computations invested in supervised learning but also reduces the memory burden for constructing random projections. In this paper, we use CS to reduce the feature dimensionality and try to prove its efficiency of speeding up text classification.

### 2.2. Compressive Sensing

CS is a novel sampling paradigm that goes against the traditional Nyquist/Shannon theorem, and it shows that a signal can be recovered precisely from only a small set of samples. The success of CS relies on two principles: sparsity and incoherence, where the former defines an *S*-sparse signal ***s*** in *R*^*N*^ with all but the *S* entries set to be zero, and the latter highlights the incoherent measure vectors {*ϕ*_*i*_ ∈ *R*^*N*^}_*i*=1_^*M*^with ***s***. The following briefly describes the CS framework.

By ordering these measure vectors in column, a measurement matrix *Φ* ∈ *R*^*M*×*N*^ is constructed as follows:

(1) $$\begin{array}{c} \Phi =\left[\begin{array}{ccc} — & \varphi_{1} & —\\ & \vdots & \\ — & \varphi_{i} & —\\ & \vdots & \\ — & \varphi_{M} & — \end{array}\right]. \end{array}$$

By using *Φ* to linearly measure *s*, we obtain the sampled vector ***y*** ∈ *R*^*M*^ by

(2) $$\begin{array}{c} y=\Phi \cdot s. \end{array}$$

We define the ratio of *M*/*N* as the subrate *R*; that is, *R* = *M*/*N*, and DR is realized by setting *R* to be less than 1, but it also becomes an ill-posed problem to find *s* from *y*. Based on the sparsity property of ***s***, this problem can be solved by an optimizing model:

(3) $$\begin{array}{c} \hat{s}=\arg\underset{s}{\min}{\left\|s\right\|}_{0}\\ \text{s}.\text{t}.y=\Phi \cdot s, \end{array}$$

where ||·||_0_ represents *l*_0_ norm to count the number of nonzero entries in *s*, and the solution $\hat{s}$ is an estimate of *s*. The incoherence between *φ*_*i*_ and ***s*** has an effect on the convergence of the solution $\hat{s}$ to the original *s*, which presents a challenge for CS, that is, how to construct incoherence measurement vectors. Fortunately, it is found that random vectors are largely incoherent with any fixed signal, so *Φ* can be produced by some random distributions, for example, Gaussian, Bernoulli, and uniform.

By performing incoherent measuring with random matrices, CS can be categorized as the random projection technology in DR. In particular, in order to enhance the robustness of recovery, CS requires *Φ* to further hold the restricted isometry property (RIP) for *S*-sparse signals. When RIP holds, *Φ* preserves the approximate Euclidean length of *S*-sparse signals, which implies that all pairwise distances between *S*-sparse signals can be well preserved in the measurement space. In text classification, the feature vectors of documents in text dataset are highly sparse, so RIP of CS can significantly reduce feature dimensionality while preserving pairwise distances between feature vectors. Superior to traditional DR methods, CS ensures less memory consumption and faster computing by SRMs. In view of the merits of CS, we explore CS features extracted by SRMs to speed up text classification.

## 3. Proposed CS-Based Text Classification

### 3.1. Framework Description

**Figure 2:**
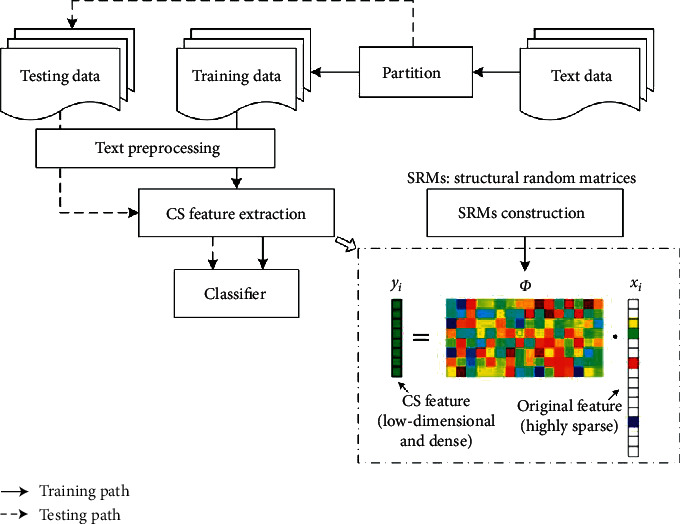
Framework of CS-based text classification.

Figure 2 presents the framework of the proposed CS-based text classification. After text preprocessing, the text dataset is divided into training dataset *P* and testing dataset *Q*, where the former is used to train classifiers, and the latter is used to evaluate the classification accuracy. The core of our work is to extract CS features to represent documents in text dataset. In CS feature extraction, we represent each document *p*_*i*_ in the training dataset *P* as the highly sparse vector *x*_*i*_ by BOW and construct an SRM *Φ* ∈ *R*^*M*×*N*^ to linearly measure *x*_*i*_, producing the CS feature vector *y*_*i*_ of *x*_*i*_. CS feature is a low-dimensional and dense vector, which can shorten the time of training classifier, especially for a large-scale text dataset. In the following parts, we describe, respectively, CS feature extraction, SRMs construction, and classifiers in detail.

### 3.2. CS Feature Extraction

We collect unigrams and bigrams from the training dataset *P* to create the vocabulary of BOW model. Unigrams are single words from *P*, and most of them occur very few times to impact classification, so we only add top *N*_1_ words from these unigrams to the BOW vocabulary. Bigrams are word pairs from *P*, and they are a good way to model negation like “not good.” The total amount of bigrams is very big, but most of them are noise at the end of frequency spectrum, so we use top *N*_2_ word pairs from these bigrams, adding them to the BOW vocabulary. In the experiment part, we set suitable *N*_1_ and *N*_2_ for different classification tasks.

After collecting unigrams and bigrams, we convert each document *p*_*i*_ in *P* into the feature vector ***x***_*i*_ in sparse representation. The BOW feature *x*_*i*_ is the frequency distribution of *p*_*i*_ on the BOW vocabulary, and its size is *N*, which is the sum of *N*_1_ and *N*_2_. All BOW features consist of a feature matrix *X* as follows:

(4) $$\begin{array}{c} X=\left[\begin{array}{ccccc} | & & | & & |\\ x_{1} & \cdots & x_{i} & \cdots & x_{L_{1}}\\ | & & | & & | \end{array}\right], \end{array}$$

where *L*_1_ is the amount of *P*. In the ordinary classification, *X* is input into the classifier to train it. Being a large size, *X* results in the curse of dimensionality; for example, when *N* and *L*_1_ are set to be 25000 and 800000, respectively, the size of *X* is 25000 × 800000, and it needs a memory of 8 × 10^10^ bytes (≈75 GB) assuming that 4 bytes encode each entry in *X*. That would lead to a heavy computational burden, so we reduce the size of *X* by CS measuring as follows:

(5) $$\begin{array}{c} Y=\left[\begin{array}{ccccc} | & & | & & |\\ y_{1} & \cdots & y_{i} & \cdots & y_{L_{1}}\\ | & & | & & | \end{array}\right]\\ =\left[\begin{array}{ccccc} | & & | & & |\\ \Phi x_{1} & \cdots & \Phi x_{i} & \cdots & \Phi x_{L_{1}}\\ | & & | & & | \end{array}\right]\\ =\Phi \cdot X, \end{array}$$

where *Φ* ∈ *R*^*M*×*N*^ is a CS measurement matrix and *Y* ∈ *R*^*M*×*L*1^ is the CS feature matrix, of which the *i*-th column *y*_*i*_ is the CS feature vector of the *i*-th document *p*_*i*_ in the training dataset *P*.

To precisely recover signals, the CS measurement matrix is required to hold RIP. In practice, a random matrix, e.g., produced by Gaussian or Bernoulli distribution, obeys RIP for *S*-sparse signal provided that

(6) $$\begin{array}{c} M\geq 4\cdot S, \end{array}$$

is satisfied [30]. *M* can be set to be far smaller than *N* since BOW features are highly sparse, so the size of *Y* can be significantly reduced. Importantly, RIP can be enforced or degraded by widening or reducing the gap between *M* and *S*; that is, when *M* is far larger than 4·*S*, the pairwise distances between *S*-sparse signals are well preserved in the CS feature space, and these pairwise distances can be destroyed when gradually reducing *M*, so the subrate *R* becomes a key factor impacting the accuracy of text classification. In the experiment part, we will evaluate the effects of different *R* values on pairwise distances between features and the accuracy of classification. In general, these random projections are dense, and a common computer does not have sufficient memory to store them, so CS-based DR is not applicable to a large-scale dataset if traditional method is used to produce the random projections. However, CS offers some measurement matrices for large-scale and real-time applications, among which the most famous is SRMs. The following describes how to construct SRMs, so as to make CS-based DR feasible for a large-scale dataset.

### 3.3. SRMs  Construction

SRM, proposed by Do et al. [28], is a known sensing framework in the field of CS. With its fast and efficient implementation, it brings some benefits to CS-based DR, for example, low complexity, fast computation, block-based processing support, and optimal incoherence. By using SRMs, with less memory consumption, the length of BOW feature can be fast and greatly reduced while holding RIP.

SRM is defined as a product of three matrices; that is,

(7) $$\begin{array}{c} \Phi =\sqrt{\frac{N}{M}}D\cdot F\cdot E, \end{array}$$

where *E* ∈ *R*^*N*×*N*^ is a random permutation matrix that uniformly permutes the locations of vector entries globally, *F* ∈ *R*^*N*×*N*^ is an orthonormal matrix constructed by popular fast computable transform, e.g., Fast Fourier Transform (FFT), Discrete Cosine Transform (DCT), Walsh-Hadamard Transform (WHT), or their block diagonal versions, *D* ∈ *R*^*M*×*N*^ is a random subset of *M* rows of the identity matrix of *N* × *N* in size to subsample the input vector, and $\sqrt{N/M}$ is a scale to normalize the transform so that the energy of the subsampled vector is almost similar to that of the input vector. By plugging (7) into (5), the matrix product *Φ*·*X* can be performed according to a sensing algorithm as shown in Algorithm 1. The SRM sensing algorithm can be computed fast; that is, the computational complexity is typically in the order of *O*(*N*) to *O*(*N*log*N*). Suppose that *F* is FFT or DCT matrix; the implementation of SRM takes *O*(*N*log*N*) operations. SRM is used to measure *L*_1_ BOW features one by one, which takes *O*(*L*_1_*N*log*N*) operations; that is, the total computational complexity of the proposed CS model is *O*(*L*_1_*N*log*N*). Compared with existing random projection techniques, SRMs not only cost less time and space complexity, but they also convert the sampled vector into a white noise-like one by scrambling the vector structure to achieve universal incoherence. Therefore, SRMs can make CS-based text classification more efficient.

### 3.4. Classifiers

Many popular classifiers can be used in our model, e.g., SVM, decision tree, AdaBoost, KNN, and Naïve Bayes. In the experiment part, these classifiers are applied and their classification accuracy is evaluated to verify the efficiency of our model. This section reviews these popular classifiers in text classification.

SVM [10] is a nonprobabilistic linear binary classifier. For a training set of points (*y*_*i*_, *l*_*i*_), where ***y***_*i*_ is the CS feature vector and *l*_*i*_ is the category of the document *d*_*i*_, we try to find the maximum-margin hyperplane that divides the points with *l*_*i*_ = 1 and *l*_*i*_ = -1. The equation of the hyperplane is as follows:

(8) $$\begin{array}{c} w^{\text{T}}y+b=0. \end{array}$$

We maximize the margin, denoted by *γ*, as

(9) $$\begin{array}{c} \underset{w,\gamma }{\max}\gamma \\ \text{s}.\text{t}.\;\forall i,\gamma \leq l_{i}\left(w^{\text{T}}y_{i}+b\right), \end{array}$$

to separate the points well. By error-correcting output codes (ECOC) model [31], SVM can also undertake multiclass classification tasks.

Decision tree [11] is a classifier model in which each node of the tree represents a test on the attribute of the data set, its children represent the outcomes, and the leaf nodes represent the final categories of the data points. The training dataset is used to form the decision tree, and the best decision has to be made for each node in the tree. The decision tree can be fast trained, but it is also extremely sensitive to small perturbations in the dataset and can be easily overfit. By cross validation and pruning, these effects can be suppressed.

AdaBoost [12] extracts a classifier from the set of weak classifiers at each iteration and assigns a weight to the classifier according to its relevance. The weight in AdaBoost for each sample is measured according to how difficult previous classifiers have found it to get it correct. At each iteration, a new classifier is trained on the training dataset, and the weights are modified based on how successfully the training sample has been classified before. Training terminates after several iterations or when all training samples are classified correctly.

KNN [13] is a nonparametric technique used for classification. Given the CS feature *y*_*i*_, KNN finds the *K*-nearest neighbors of *y*_*i*_ among all CS features in the training dataset and gives the category candidate a score based on the labels of the *K* neighbors. The similarity between *y*_*i*_ and its neighbor can be the score of the category of the neighbor features. After sorting the score values, KNN decides which category the candidate falls into with the highest score from *y*_*i*_. KNN is easy to implement and adapts to any kind of feature space. It can also handle multiclass cases. The performance of KNN depends on finding some meaningful distance functions, and it is limited by data storage when finding the nearest neighbors for large search problems.

Naïve Bayes [14] has been widely used for text classification, and it is a generative model based on Bayes theorem. This model assumes that the value of a particular feature is independent of the value of any other feature. The proposed CS model is on the assumption that any entry in a CS feature vector is independent of other entries. Given a to-be-tested CS feature ***y***, its category is predicted as follows:

(10) $$\begin{array}{c} \hat{l}=\arg\underset{l}{\max}p\left(\left.l\right|y\right). \end{array}$$

According to Bayes inference, we see that

(11) $$\begin{array}{c} p\left(\left.l\right|y\right)\propto p\left(l\right)\prod_{m=1}^{M}p\left(\left.y_{m}\right|l\right), \end{array}$$

where *y*_*m*_ is the *m*-th entry in the CS feature *y*. The probabilities *p*(*l*) and *p*(*y*_*m*_|*l*) can be estimated by maximum likelihood on the training dataset.

## 4. Experimental Results

### 4.1. Dataset and Setting

**Figure 3:**
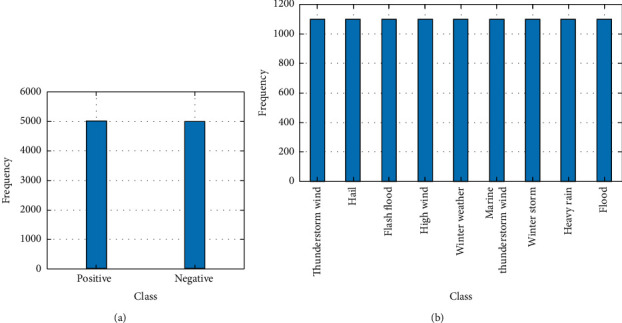
Statistics of the Twitter sentiment and weather report datasets after balancing. (a) Twitter sentiment dataset. (b) Weather report dataset.

We conduct experiments on two datasets, one for a binary classification task and the other for a multiclass classification task. For the binary classification task, we use the Twitter sentiment dataset, which was crawled and labeled positive or negative. For the multiclass classification task, we use the weather report dataset that contains a text description and category labels for each event including thunderstorm wind, hail, flash flood, high wind, and winter weather. The classes of two datasets are imbalanced, especially for weather report dataset. To avoid the effects of imbalance on classification accuracy, the two datasets are preprocessed to make their classes balanced; i.e., for Twitter sentiment dataset, we randomly remove some positive and negative observations and make each class having 10000 observations; for weather report dataset, we delete the classes with few observations, and 9 classes remain: thunderstorm wind, hail, flash flood, high wind, winter weather, Marine Thunderstorm Wind, Winter Storm, Heavy Rain, and Flood, among which one has 1000 observations.  Figure 3 presents the statistics of Twitter sentiment dataset and weather report dataset after balancing. For any dataset, 20% of observations in each class are set aside at random for testing. In feature extraction, we first do some preprocessing on documents in two datasets including the following: (1) tokenize the documents; (2) lemmatize the words; (3) erase punctuation; (4) remove a list of stop words such as “and,” “of,”, and “the”; (5) remove words with 2 or fewer characters; (6) remove words with 15 or more characters. Then, for both datasets, we, respectively, collect the top 8000 unigrams and 10000 bigrams from the training set to construct the BOW vocabulary, i.e., *N*_1_ = 8000 and *N*_2_ = 10000, and represent each training observation as the BOW feature vector with length of *N* being 18000. Finally, by setting different subrates, the SRMs are used to measure the BOW feature vectors, and the corresponding CS feature vectors are produced. We train different classifiers on the BOW-based and CS-based training sets, respectively, tune parameters by cross validation, and evaluate these classifiers on the test sets. Due to the random partition of dataset, the training and testing are repeated five times, and the mean testing accuracy is used as the evaluation metrics.

The experimental settings are as follows. To evaluate the effects of different SRMs on feature distance and classification accuracy, we construct five SRMs by using transform matrices *F* including DCT, FFT, Block DCT, Block WHT, and Block Gaussian, in which the latter three are block diagonal matrices, of which the diagonal elements are DCT and WHT and Gaussian matrices with the size of 32 × 32. We use six classifiers including SVM, decision tree, AdaBoost, KNN, and Naïve Bayes to evaluate the classification accuracy of our model and compare the proposed CS model with the three DR methods: PCA [17], ICA [18], and NMF [19]. The subrate *R* is set to be between 0.1 and 0.6, and it is preset parameter, which is used to decide the length of CS feature vector. All of the experiments are conducted under the following computer configuration: Intel(R) Core (TM) i7 @3.30 GHz CPU, 8 GB, RAM, Microsoft Windows 7 64 bits, and MATLAB Version 9.6.0. (R2019a). The datasets and experimental codes have been downloaded from SIGMULL Team Website: http://www.scholat.com/showTeamScholar.html?id=1234&changeTo=Ch&nav=4.

### 4.2. Effects of SRMs

**Figure 4:**
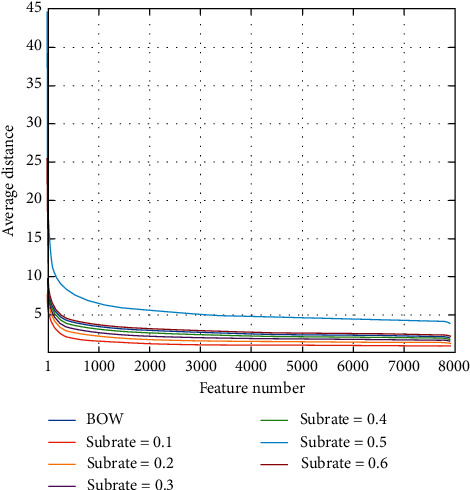
Average distances between any BOW or CS feature and others on multiclass classification dataset at different subrates when using Block DCT matrix. BOW denotes average distances between any BOW feature and others. These average distances are sorted in a descending order.

Feature distance measures the similarity between any two documents, which has a significant impact on training accuracy. If the features output by DR can well preserve their pairwise distances in original space, DR suppresses the loss of training accuracy; therefore, we evaluate the effects of SRMs on pairwise distances between text features. In the training set *P*, the average distance between the *i*-th BOW or CS feature and others is computed as follows:

(12) $$\begin{array}{c} {\text{dist}}_{i}^{\text{BOW}}=\frac{1}{L_{1}}\sum_{j=1}^{L_{1}}{\left\|x_{i}-x_{j}\right\|}_{2}, \end{array}$$

(13) $$\begin{array}{c} {\text{dist}}_{i}^{\text{CS}}=\frac{1}{L_{1}}\sum_{j=1}^{L_{1}}{\left\|y_{i}-y_{j}\right\|}_{2}, \end{array}$$

where *x*_*i*_ and *y*_*i*_ are, respectively, the *i*-th BOW and CS feature vector in *P* and *L*_1_ is the amount of *P*. We select Block DCT as the core of SRM and use (12) and (13) to compute the average distance of each BOW and CS feature as shown in Figure 4. We can see that the tendencies of all distance curves are similar, and the curve of CS features trends closer to that of BOW features as the subrate increases, which indicates that the pairwise distances between BOW features correspond to those between CS features. To measure the distance differences between BOW and CS features, we compute the Mean Square Error (MSE) between the average distances of BOW and CS features as follows:

(14) $$\begin{array}{c} {\text{MSE}}_{\text{dist}}=\frac{1}{L_{1}}\sum_{i=1}^{L_{1}}{\left({\text{dist}}_{i}^{\text{BOW}}-{\text{dist}}_{i}^{\text{CS}}\right)}^{2}. \end{array}$$

Table 1 presents the MSEs on multiclass classification dataset when using different subrates and SRMs. It can be seen from Table 1 that all SRMs provide similar MSEs at any subrate; e.g., the average MSE of each SRM at all subrates is about 11.00, and the MSEs of SRMs decrease as the subrate increases; e.g., the MSE of DCT is 18.78 at the subrate of 0.1, and it is reduced to 5.92 at the subrate of 0.6. These MSE results indicate that SRMs can preserve the approximate pairwise distances between BOW features in the CS feature space.

Then, we select SVM as the classifier in our model and evaluate the effects of SRMs on classification accuracy. With different SRMs, the accuracies of SVM classifier on binary and multiclass classification datasets are presented in Table 2. It can be seen that all SRMs provide similar accuracies in most cases at any subrate; e.g., with all subrates considered, the average accuracies of SRMs range from 0.7121 to 0.7203 on binary classification dataset, and similar results are obtained on multiclass classification dataset. We also see that the accuracy is gradually improved for any SRM as the subrate increases. The above results indicate that the selection of SRMs has little impact on classification accuracy, and the subrate is a key factor in controlling the accuracy. Therefore, any SRM can be used in our model, and we need to consider the balance between accuracy and subrate in practice.

### 4.3. Evaluation on Classifiers

**Figure 5:**
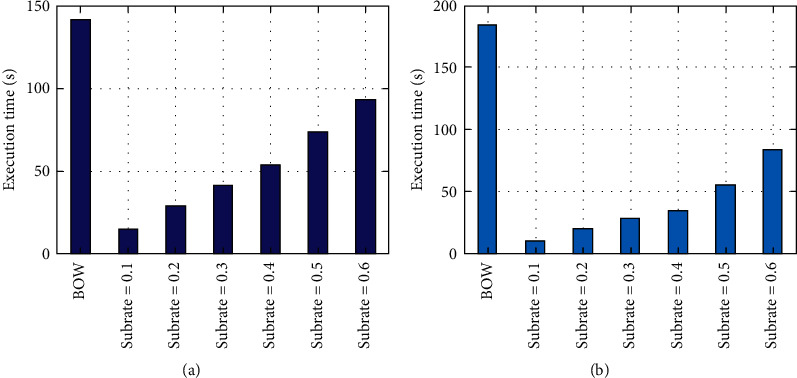
Average training time (s) on all classifiers driven by BOW and CS features for binary and multiclass classification tasks when SRM is Block DCT. (a) Binary classification. (b) Multiclass classification.

To verify the validity of CS, we have compared CS features and BOW features in terms of the accuracies and training time of different classifiers driven by them. The Block DCT is selected as SRM, and the accuracy results are presented in Table 3. It can be seen that, for binary classification, the accuracies of classifiers driven by the CS features go up with the increase of subrate. Though lower than those with BOW feature when the subrate is small, they quickly catch up; e.g., for SVM, the CS feature overtakes the BOW feature when the subrate is 0.3 and outperforms it thereafter. All the classifiers considered, the average accuracy by the CS features is also comparable with that by BOW feature. The same result can be obtained for multiclass classification. As for the training time in Figure 5, whether it is binary or multiclass classification, the CS feature costs far less than the BOW feature, especially when the subrate is small.  Table 4 presents average accuracy, precision, recall, and F_1_ on all classifiers for binary classification dataset. It can be seen that the precision, recall, and F_1_ by CS features at any subrate are similar to those by BOW features, which indicates that the classification accuracy is reliable for CS features. From the above results, it can be concluded that CS speeds up the training of classifiers while providing the accuracies that can match the BOW feature.

### 4.4. Comparisons on DR Methods

We compare the performance of the proposed CS model with that of some popular DR methods including PCA, ICA, and NMF. PCA learns all principal components from the training set, and, according to the preset subrate, selects part of principal components to construct the transform matrix. ICA and NMF learn their transform matrices at different subrates by numerical iterative algorithms, and their maximum numbers of iterations are both set to be 20 in order to keep the execution time at a moderate level. We use each of the above transform matrices to project all training and testing observations onto a low-dimension space. The proposed CS model uses Block DCT to reduce the dimensionalities of observations at different subrates.  Table 5 presents the average accuracies of all classifiers for binary and multiclass classification datasets when using different DR methods. We can see that the proposed CS model obtains higher accuracies than PCA, ICA, and NMF at any subrate for both binary and multiclass classification tasks. The proposed CS model is more stable, and its accuracy increases gradually as the subrate increases, but the accuracies of PCA, ICA, and NMF float up and down as the subrate increases; for example, for binary classification, the accuracy of PCA is 0.6221 at the subrate of 0.1. However, when the subrate is raised to 0.6, the accuracy drops to 0.6091.  Table 6 presents the execution time of different DR methods on binary and multiclass classification datasets when using different subrates. PCA learns all principal components, so its execution time does not vary as the subrate increases, and it costs 387.75 s and 275.49 s for binary classification and multiclass classification, respectively. At the preset subrate, ICA and NMF determine the final dimensionalities of observations and learn the corresponding transform matrices, so their execution time increases as the subrate increases; e.g., for binary classification, NMF costs 187.33 s at the subrate of 0.1 and costs 2201.12 at the subrate of 0.6. The accuracies of ICA and NMF can be improved by increasing iteration times, but their execution time can also increase dramatically. Compared with PCA, ICA, and NMF, the proposed CS model costs less execution time; e.g., for binary classification, CS costs only 3.32 s and 4.63 s at the subrates of 0.1 and 0.6, respectively. From the above results, it can be concluded that the proposed CS model obtains higher accuracy with less execution time when compared with PCA, ICA, and NMF. Therefore, the proposed CS model is a reliable DR method.

## 5. Conclusion

In this paper, we develop a CS-based model for text classification tasks. Traditionally, the BOW features are extracted from the text dataset, and they are the highly sparse representations with a huge dimensionality. It costs a lot to train classifiers by using BOW features. By using the incoherent measuring of CS, we greatly reduce the dimensionality of BOW features, and, at the same time, the RIP of CS ensures that the pairwise distances between BOW features are well preserved in a low-dimensional CS feature space. CS also provides the SRMs that are fast computable with low memory consumption. In the proposed model, different SRMs are constructed to linearly measure BOW features at a preset subrate, generating the CS features that are used to train the classifiers. Experimental results show that the proposed CS model provides a comparable classification accuracy with the traditional BOW model and significantly reduces the space and time complexity required by a large-scale dataset training.

## Figures and Tables

**Algorithm 1:**
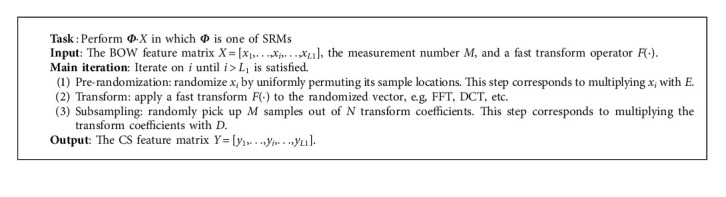
Flow of SRM sensing algorithm.

**Table 1:** MSEs between average distances of CS and BOW features on multiclass classification dataset when using different subrates and SRMs.

Subrate *R*	SRMs				
	DCT	FFT	Block DCT	Block WHT	Block Gaussian
0.1	18.78	18.81	18.38	18.48	18.62
0.2	14.37	14.40	14.38	14.08	14.51
0.3	11.39	11.39	11.17	11.11	11.78
0.4	9.14	9.12	9.018	9.01	9.33
0.5	7.36	7.34	7.19	7.21	7.33
0.6	5.92	5.91	5.96	5.90	6.03
Avg.	11.16	11.16	11.02	10.96	11.27

**Table 2:** Accuracies of SVM classifier associated with different SRMs on binary and multiclass classification datasets at different subrates.

Subrate *R*	SRMs				
	DCT	FFT	Block DCT	Block WHT	Block Gaussian
Binary classification					
0.1	0.6955	0.7220	0.6975	0.6880	0.6930
0.2	0.7185	0.7135	0.7135	0.7200	0.7055
0.3	0.7195	0.7140	0.7285	0.7215	0.7125
0.4	0.7285	0.7190	0.7265	0.7170	0.7185
0.5	0.7235	0.7195	0.7290	0.7270	0.7145
0.6	0.7255	0.7290	0.7265	0.7280	0.7285
Avg.	0.7185	0.7195	0.7203	0.7169	0.7121

Multiclass classification					
0.1	0.8590	0.8575	0.8358	0.8444	0.8227
0.2	0.8616	0.8606	0.8651	0.8636	0.8585
0.3	0.8651	0.8737	0.8666	0.8737	0.8606
0.4	0.8686	0.8702	0.8712	0.8747	0.8712
0.5	0.8712	0.8732	0.8767	0.8691	0.8757
0.6	0.8747	0.8782	0.8803	0.8732	0.8762
Avg.	0.8668	0.8689	0.8660	0.8665	0.8609

**Table 3:** Accuracies of different classifiers driven by BOW and CS features on binary and multiclass classification datasets when SRM is Block DCT.

Classifier	BOW feature	Subrate *R* for CS feature					
		0.1	0.2	0.3	0.4	0.5	0.6
Binary classification							
SVM	0.7220	0.6975	0.7135	0.7285	0.7265	0.7290	0.7265
Decision tree	0.6235	0.6365	0.6395	0.6460	0.6355	0.6465	0.6485
AdaBoost	0.7060	0.7020	0.6975	0.7075	0.7035	0.7020	0.7110
KNN	0.6040	0.5955	0.6120	0.6200	0.6140	0.6145	0.6125
Naïve Bayes	0.7275	0.7035	0.7130	0.7125	0.7170	0.7200	0.7150
Avg.	0.6766	0.6670	0.6751	0.6829	0.6793	0.6824	0.6827

Multiclass classification							
SVM	0.8732	0.8358	0.8651	0.8666	0.8712	0.8767	0.8803
Decision tree	0.8560	0.8454	0.8434	0.8510	0.8520	0.8525	0.8530
AdaBoost	0.7777	0.7535	0.7737	0.7732	0.7813	0.7808	0.7818
KNN	0.8252	0.8080	0.8146	0.8207	0.8242	0.8257	0.8252
Naïve Bayes	0.7737	0.7373	0.7404	0.7464	0.7429	0.7424	0.7454
Avg.	0.8212	0.7960	0.8074	0.8116	0.8143	0.8156	0.8171

**Table 4:** Average accuracy, precision, recall, and F_1_ on all classifiers for binary classification dataset when SRM is Block DCT.

Metrics	BOW feature	Subrate *R* for CS feature					
		0.1	0.2	0.3	0.4	0.5	0.6
Accuracy	0.6766	0.6670	0.6751	0.6829	0.6793	0.6824	0.6827
Precision	0.6564	0.6658	0.6674	0.6722	0.6694	0.6670	0.6694
Recall	0.6817	0.6671	0.6775	0.6866	0.6824	0.6871	0.6864
F_1_	0.6679	0.6664	0.6723	0.6790	0.6756	0.6766	0.6774

**Table 5:** Average accuracies of all classifiers for binary and multiclass classification datasets when using different DR methods.

DR method	Subrate *R*					
	0.1	0.2	0.3	0.4	0.5	0.6
Binary classification						
PCA	0.6221	0.6236	0.6206	0.6154	0.6222	0.6091
ICA	0.5754	0.5830	0.5862	0.5974	0.5903	0.6009
NMF	0.5926	0.6127	0.6193	0.6067	0.6157	0.6000
CS	0.6670	0.6751	0.6829	0.6793	0.6824	0.6827

Multiclass classification						
PCA	0.7253	0.7213	0.7019	0.6845	0.6822	0.6726
ICA	0.4938	0.5170	0.5305	0.5448	0.5455	0.5479
NMF	0.7112	0.7080	0.7123	0.7123	0.7096	0.7063
CS	0.7960	0.8074	0.8116	0.8143	0.8156	0.8171

Note that SRM in CS is Block DCT.

**Table 6:** Execution time (s) of different DR methods on binary and multiclass classification datasets when using different subrates.

DR method	Subrate *R*					
	0.1	0.2	0.3	0.4	0.5	0.6
Binary classification						
PCA	384.75	384.75	384.75	384.75	384.75	384.75
ICA	369.72	3094.00	17259.27	34511.16	35281.73	50355.25
NMF	187.33	456.67	1169.65	1873.32	2481.44	2201.12
CS	3.32	3.64	3.92	4.19	4.58	4.63

Multiclass classification						
PCA	275.49	275.49	275.49	275.49	275.49	275.49
ICA	188.77	382.82	990.19	6592.11	10829.64	20559.64
NMF	159.21	327.14	652.83	1239.35	1529.07	2358.88
CS	3.10	3.77	3.94	4.03	4.25	4.41

Note that SRM in CS is Block DCT.

## Data Availability

The datasets and experimental codes have been downloaded from SIGMULL Team Website: http://www.scholat.com/showTeamScholarEn.html?id=1234&changeTo=En&nav=4.
